# Interspecific Neighbor Interactions Promote the Positive Diversity-Productivity Relationship in Experimental Grassland Communities

**DOI:** 10.1371/journal.pone.0111434

**Published:** 2014-10-28

**Authors:** Yuhua Zhang, Yongfan Wang, Shixiao Yu

**Affiliations:** Department of Ecology, School of Life Sciences/State Key Laboratory of Biocontrol, Sun Yat-sen University, Guangzhou, China; University of Oxford, United Kingdom

## Abstract

Because the frequency of heterospecific interactions inevitably increases with species richness in a community, biodiversity effects must be expressed by such interactions. However, little is understood how heterospecific interactions affect ecosystem productivity because rarely are biodiversity ecosystem functioning experiments spatially explicitly manipulated. To test the effect of heterospecific interactions on productivity, direct evidence of heterospecific neighborhood interaction is needed. In this study we conducted experiments with a detailed spatial design to investigate whether and how heterospecific neighborhood interactions promote primary productivity in a grassland community. The results showed that increasing the heterospecific: conspecific contact ratio significantly increased productivity. We found there was a significant difference in the variation in plant height between monoculture and mixture communities, suggesting that height-asymmetric competition for light plays a central role in promoting productivity. Heterospecific interactions make tall plants grow taller and short plants become smaller in mixtures compared to monocultures, thereby increasing the efficiency of light interception and utilization. Overyielding in the mixture communities arises from the fact that the loss in the growth of short plants is compensated by the increased growth of tall plants. The positive correlation between species richness and primary production was strengthened by increasing the frequency of heterospecific interactions. We conclude that species richness significantly promotes primary ecosystem production through heterospecific neighborhood interactions.

## Introduction

Understanding the role of biodiversity in promoting ecosystem functions, such as primary production, is critically important to biodiversity conservation and ecosystem management. Empirical studies showed that diversity-productivity relationships can take various forms [1], [2], [3], [4]. However, positive relationships, the productivity increases with diversity, have been overwhelmingly documented by many manipulative biodiversity experiments [5], [6], [7], [8], [9], [10], [11].

Complementarity and selection effects are the two primary mechanisms used to interpret the positive diversity-productivity relationships [12]. The complementarity effect results from resource partitioning, natural enemy regulation or facilitative interactions between species in mixture communities [7], [13], [14], [15], while the selection effect is due to shifts in dominance driven by heterospecific competition [16], [17], [7]. Although the selection effect does play a role, its effect is variable and its importance often tends to decrease over time, leaving the complementarity effect as the main factor explaining the positive effect of biodiversity on ecosystem functioning [12], [13], [8], [9], [18], [19]. A critical, but largely unresolved question is: what are the biological mechanisms that drive the complementarity effect and how do they enhance productivity in mixture communities?

The main difference between mixtures and monocultures is that the former are subject to heterospecific interaction effects while the latter experience only conspecific competition. Heterospecific interactions must produce more improvements on average than conspecific interactions when generating positive effects on ecosystem functioning. They must also ensure the coexistence of the species present, which links the biodiversity–ecosystem functioning and species coexistence theories, albeit in complex ways [20], [15]. Therefore, resource partitioning and facilitation have often been used to explain the positive effects of biodiversity on ecosystem functioning, whether through belowground processes that lead to enhanced soil nutrient utilization [21], [22], [23] or by reduced competition for light [24], [25]. Variation in traits, such as plant height, plant architecture effects on light competition, and rooting depth and spread for water or nutrient utilization, are essential complementarity effects that can produce overyielding in mixtures.

Heterospecific competition can further increase heterospecific trait variation in mixtures, either through niche shifts in the presence of heterospecific competitors or by differential growth, thereby increasing the potential for complementarity between species and overyielding in mixtures. For instance, under light competition, short plants may become shorter while tall plants become taller in mixtures, compared to monocultures, so that the loss of biomass by the inferior species is compensated by the biomass gain in the superior species. Consequently, both negative (mainly for short species) and positive (mainly for tall species) neighborhood interspecific interactions can be observed in a mixture, e.g., as in the case of soil nitrate competition [21].

Experiments have been conducted to test for the effect of interspecific interactions on biomass [26], [27], [21], [25], [28]. Such experiments are commonly designed to compare biomass in plots where the seeds have been sown by random broadcasting versus that of aggregated sowing. Most results have shown that biomass in randomly sown plots is higher than that in aggregated sown plots due to the more efficient use of soil nutrients or light in the former case, presumably due to the higher frequency of heterospecific interactions [24], [21], [23], [25], [28], [29]. However, in these studies, the plots are considered the basic experimental unit and sowing method (dispersed versus aggregated) as a treatment factor. No data on the frequency of heterospecific neighbor interactions within the plots were collected. Such data are essential for directly inferring the effects of heterospecific interactions [6].

We conducted an intensive study to investigate the effect of interspecific interactions on plant growth in grassland communities and to show how that gives rise to a positive diversity-productivity relationship. The study consisted of two complementary experiments. The first experiment was designed to measure the growth of plants in each grid within a series of plots that varied in sowing density, species richness and frequency of heterospecific interactions. The second experiment enhanced the first one by adding more diversity levels. Based on these, we first modeled the effects of species richness and the frequency of heterospecific interactions on plant growth across the plots. We then tested the hypothesis that the positive effect of heterospecific interactions on productivity in grassland communities was driven by height-asymmetric competition for light in mixture plots.

## Materials and Methods

### Ethics Statement

The study site is maintained by Heerkou Town, Fengkai County, Guangdong Province, China. The site was leased to Sun Yat-sen University from 2009 to 2013 for conducting the grassland experiments reported in this study. Our study did not involve any damage to land resources and no specific permissions were required for this research. Our experiments didn’t contain any treatments with chemical addition. The seeds of all plant species used in our experiments were collected from the area around the experimental site, and these plants were neither endangered nor protected species.

### Experimental Design

**Figure 1:**
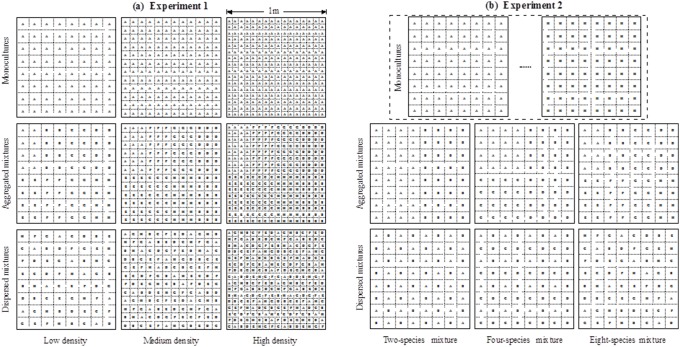
Experimental esign of the two experiments. (*a*) The first experiment comprised 9 blocks involving 8 species. Each block consisted of 36 plots (1×1 m in size). Illustrated here is one block but only shows 9 plots. The other 27 plots are not shown, including 21 monoculture plots for other 7 species (each being sown at low, medium and high density) and six mixcultures (i.e., the two spatial configurations for the 3 density levels, the same as the second and third rows). The aggregated and dispersed mixcultures both had the same 8 species but different spatial configurations. (*b*) The second experiment comprised 5 blocks involving 8 species. Each block consisted of 32 1×1 m plots. Illustrated here is one block but only shows 8 plots. The other 24 plots are not shown, including 6 monoculture plots for the other 6 species and 3 replicates for each of the six mixcultures (i.e., the two spatial configurations for the 3 diversity level). In both experiments, blocks were separated by 2 m walkways, and plots were separated 1 m apart.

The experimental site was located in a subtropical arable field near the Heishiding Nature Reserve (111°53′ E, 23°26′ N), Guangdong Province, China [30]. The soil was a ferralosol. The first experiment was established in 2009 and comprised of nine blocks involving eight species (Fig. 1*a*). Each block consisted of 36 plots (each 1 m×1 m) that varied in sowing density, number of species and spatial pattern. Sowing density across plots within a block varied from low (64 grids/plot), to medium (144 grids/plot) to high (256 grids/plot) (Fig. 1*a*). There were two diversity levels (monoculture and 8 species mixtures). Each of the eight species had three monocultures with densities varying from low, medium to high in each of the nine blocks, which made a total of 24 monoculture plots per block. In addition, there were two spatial patterns (aggregated and dispersed) for each density level (Fig. 1*a*) and the aggregated and dispersed patterns were replicated twice, making a total of 12 plots (i.e., 2 spatial patterns×3 densities×2 replicates). The dispersed spatial pattern was designed to maximize heterospecific interactions. In total, there were 324 plots across the nine blocks for this experiment. All of the 324 plots were hand-seeded in February 2009 and 10 seeds of each species were sown in each grid of a plot. In total, 640, 1440, 2560 seeds, respectively, were sown in each plot in order to represent the three density treatment levels. The eight species used in this experiment were all native species and were randomly selected from the local area. They were *Ambrosia artemisiifolia* (Compositae), *Urena lobata* (Malvaceae), *Triumfetta rhomboidea* (Tiliaceae), *Bidens pilosa* (Compositae), *Mosla dianthera* (Labiatae), *Pennisetum alopecuroides* (Gramineae), *Epimeredi indica* (Labiatae) and *Corchorus capsularis* (Tiliaceae), which were respectively referred to as A, B, C, D, E, F, G, H (Fig. 1*a*).

The second experiment was established in a nearby arable field in 2010. It had five blocks. Each block comprised of 32 plots, including eight monocultures (each for one of the eight species) and six mixtures that had two spatial patterns (aggregated and dispersed) and three diversity levels (two, four and eight species) (Fig. 1*b*). Each of the six mixtures was replicated four times. In total, there were 160 plots across the five blocks for this experiment. In contrast to the first experiment, the sowing density per plot was fixed at 64 grids, while the diversity levels were either one, two, four or eight species (Fig. 1*b*). Another difference in this experiment was that the eight species in each plot were not fixed but were randomly selected from a species pool of 15 species. The species pool included all the eight species included in the first experiment plus seven new species: *Chenopodium ambrosioides* (Chenopodiaceae), *Cassia occidentalis* (Leguminosae), *Cassia tora* (Leguminosae), *Keiskea australis* (Labiatae), *Lespedeza cuneata* (Leguminosae), *Elsholtzia ciliata* (Labiatae) and *Sida acuta* (Malvaceae). All 160 plots were hand-seeded in April 2010 and 10 seeds per species were sown in each plot grid.

### Biomass Harvesting

The plots were weeded monthly during the study, but the weeds were not measured and were thus excluded from the study. The plots were harvested at the end of the growing season (late September in 2009 and 2010 for the respective experiments). For experiment I, the height of the tallest plant in each plot grid was measured. The plants in each grid were then gently removed, and the soil was shaken and washed off. The roots were separated from the shoots. The clipped shoots for each grid were weighed after oven drying at 80°C for 48 hours. The clipped roots for each species were combined across all the grids for each plot and weighed after oven drying at 80°C for 48 hours. This meant that in this experiment both the aboveground and belowground biomasses were measured for each plot. This experiment consisted of two diversity levels (monocultures versus eight species mixtures). All plants in one plot were dead at before harvesting and this plot was excluded from analysis. At the end, experiment I only had 323 plots (see data in Table S1).

For experiment II, aboveground biomass for each species was harvested for each plot (belowground biomass was not measured). The plants of each species in each plot were weighed after oven drying at 80°C for 48 hours. This experiment had four species richness levels (one, two, four, and eight species) compared to the first experiment that had two species richness levels (one and eight species). This experiment complemented the first one by showing how productivity changed with species richness at different levels of heterospecific interactions in the aggregated versus dispersed plot designs.

### Data Analysis

We modeled the effects of species richness and the frequency of heterospecific neighbor interactions on plant growth. The analysis was based on the biomass data measured at the plot level for both experiment I (for above and belowground biomass) and experiment II (for aboveground biomass only). For experiment I (Fig. 1*a*), there were three density levels (64, 144 and 256 grids) and two diversity levels (one versus eight species). Spatial patterns in this experiment were either monocultures, aggregated mixtures or dispersed mixtures. Monocultures had no heterospecific interactions, aggregated mixtures had an intermediate heterospecific interaction frequency and dispersed mixtures had the greatest heterospecific interaction frequency.

Multiple linear regression (i.e., ANOVA) can be used to model biomass by considering the frequency of heterospecific interactions, species richness and plant density as explanatory variables. A more efficient approach is to treat plant density as a grouping factor and use a mixed effect model to show biomass changes (although both models led to the same conclusions). The mixed effect model was:
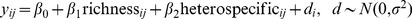
(1)where *y_ij_* is biomass at density *i* and plot *j*, heterospecific*_ij_* is the average number of heterospecific neighbors around a focal plant and *d_i_* is plant density as a random effect. The experimental design does not permit modeling the interactive term between *richness* and *interspecific* because when *richness* = 1, no aggregated or dispersed mixtures exist. This mixed effect model (1) was used to model both the aboveground and belowground biomass in experiment I, in terms of species richness and heterospecific interactions. All the data were Box-Cox transformed to ensure normality (see the transformed data were enclosed in Table S2). Because a multiple regression is a “partial” regression, *β*_2_ describes the effect of heterospecific interactions on biomass, given that the effects of species richness and density are already accounted for.

In a similar manner to experiment I, experiment II had the same three levels of spatial patterns (monocultures, aggregated and dispersed mixtures) (Fig. 1*b*). Different from experiment I, it only had one density level (64 grids) for all 160 plots and four species richness levels (one, two, four or eight species). The model was:
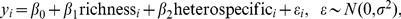
(2)where *y_i_* is biomass in plot *i*. The biomass was Box-Cox transformed to achieve normality.

To test the hypothesis that height-asymmetric competition for light is responsible for overyielding in mixture plots, we compared the root/shoot biomass ratio, plant height and height variance for monocultures, aggregated mixtures and dispersed mixtures. If competition for light is a determining factor, we should expect the variation in height to increase from monocultures to aggregated and finally to dispersed mixtures, while the average height remains largely unchanged. We should also see that heterospecific competition would make tall plants grow taller and for short plants to become smaller in mixture plots compared to monocultures.

Statistical program R (http://www.r-project.org/) was used to analyze the data. Package “nlme” was used to estimate model (1).

## Results

**Figure 2:**
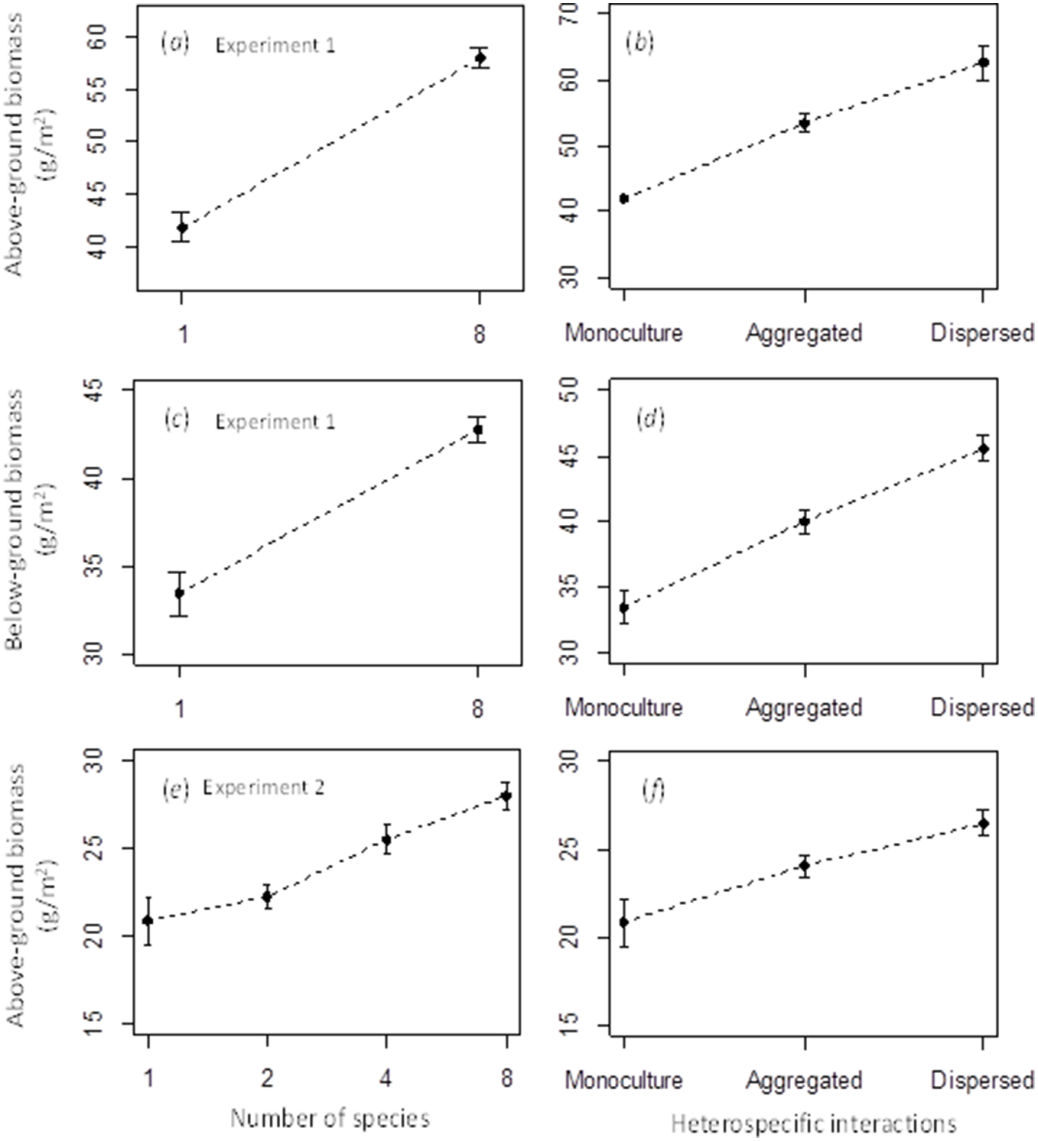
Effects of the number of species and heterospecific interactions on above- and belowbiomass. Mean ± SE for the Box-Cox transformed above-ground biomass (*a* and *b*) and below-ground biomass (*c* and *d*) of experiment I and for the Box-Cox transformed above-ground biomass for experiment II (*e* and *f*). The *x*-axis label on the left column is the number of species. Experiment I had 2 richness levels (1 and 8 species). Experiment II had four richness levels (1, 2, 4 and 8 species).

For the first experiment, highly significant positive effects for both diversity and the frequency of interspecific interactions on aboveground biomass were demonstrated with the mixed effect model (1), while the effects of diversity and the frequency of interspecific interaction on belowground biomass were weaker (Table 1). The relationships between species richness and biomass and between the frequency of heterospecific interactions and biomass for experiment I are shown in Fig. 2. It is clear both relationships were positive.

**Table 1:** Results of the mixed effects model for experiment I and of multiple regression model for experiment II for testing the effects of species richness and interspecific interactions on plot biomass for each experiment.

Experiment	*β_0_* (intercept)	*β*_1_(species richness)	*β_2_*(interspecific interactions)
Experiment I: Above-groundbiomass	40.579±2.351***	1.351±0.416***	4.932±1.630***
Experiment I: Below-groundbiomass	32.577±1.921***	0.828±0.387*	2.644±1.516^†^
Experiment II: Above-groundbiomass	19.811±0.835***	0.843±0.192***	0.766±0.331*

The Box-Cox transformed biomass were used in the models. The values (± SE) are regression coefficients. ***for *P*<0.001, **for *P*<0.01, *for *P*<0.05, and ^†^for *P*<0.1.

**Figure 3:**
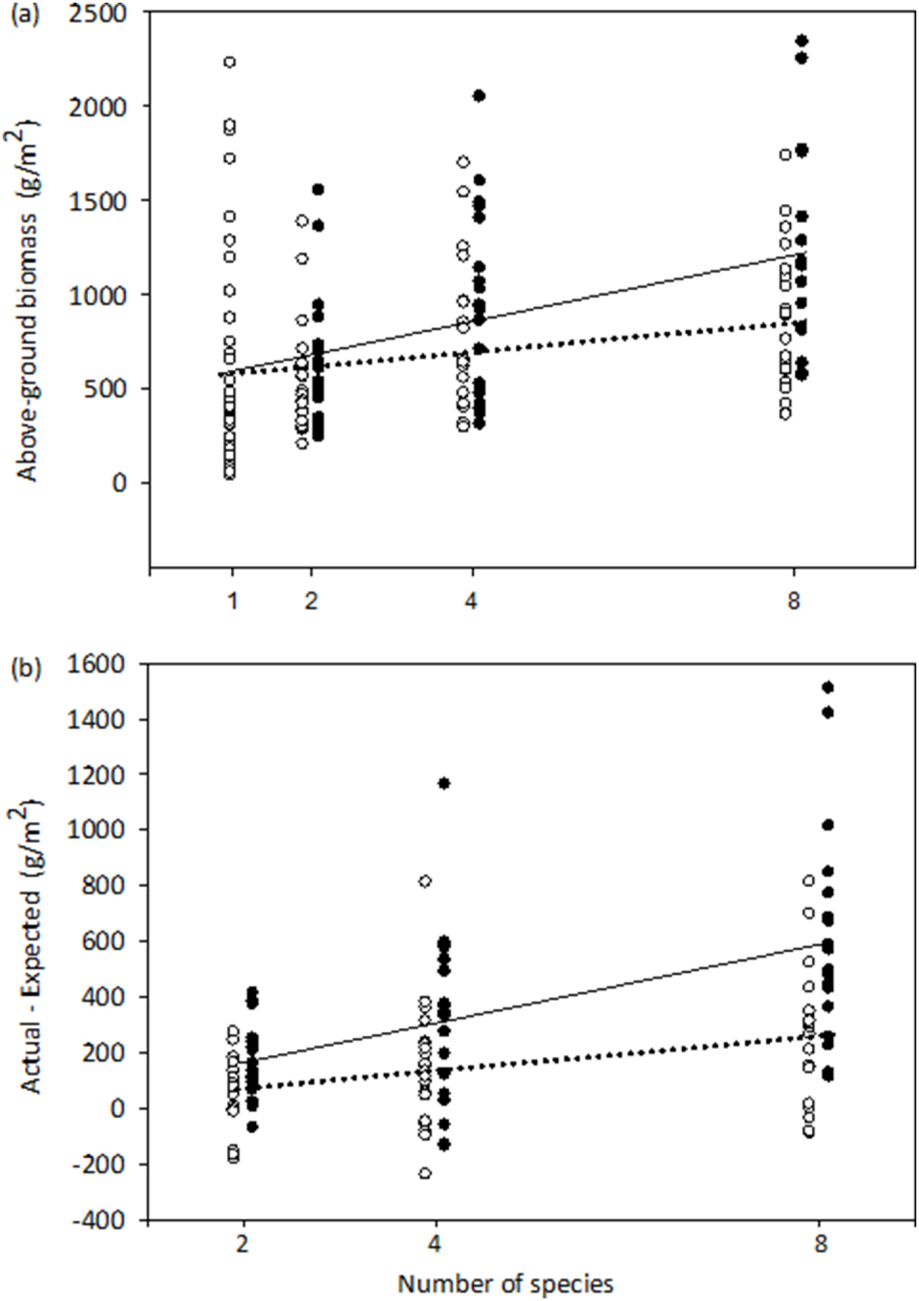
Overyielding and the magnitude of complementary effects. (*a*) Linear relationships between aboveground biomass and species richness for aggregated (dashed lines and open circles) and dispersed mixtures (solid lines and filled circles) of experiment II. (*b*) Difference in aboveground biomass between the observed mixture plots and the mean monoculture biomass of all species across diversity gradients for experiment II. This difference measures overyielding and the degree of difference indicates the degree of complementarity effects. Dashed lines and open circles refer to plots with aggregated mixtures, and solid lines and filled circles refer to dispersed plots.

For the second experiment, the results of the multiple linear regression model (2) showed that both diversity and the frequency of heterospecific neighbor interactions significantly affected plot biomass (Table 1). The positive effects of species richness and heterospecific interactions on biomass are shown in Fig. 2. We can also show how biomass changed with species richness for the aggregated and dispersed mixtures by comparing the observed biomass of the mixture plots against the mean biomass averaged from all the monoculture plots (Fig. 3). For the aggregated pattern, there was a positive relationship between species richness and aboveground biomass (*r* ^2^ = 0.05, *P* = 0.026) (Fig. 3*a*). This positive relationship became stronger in the dispersed pattern (*r* ^2^ = 0.17, *P*<0.0001), although the slope of this relationship is only marginally significantly higher than that of the aggregated pattern (*t* = −1.782, *P* = 0.0763; Fig. 3*a*). Figure 3*b* shows the difference between the observed biomass for mixture plots (aggregated and dispersed) and the mean biomass averaged from all the monoculture plots. For the aggregated mixtures, the difference did not significantly increase with species richness (*r* ^2^ = 0.04, *P* = 0.123) although there is a noticeable trend of increase. For the dispersed mixtures, the difference significantly increased with species richness (*r* ^2^ = 0.19, *P* = 0.0006). The slope of the linear relationship for the dispersed pattern was significantly (marginally) higher than the slope for the aggregated pattern (*t* = −1.917, *P* = 0.0577; Fig. 3*b*). These results indicated that increasing the heterospecific interaction frequency strengthened the positive diversity-productivity relationship.

**Figure 4:**
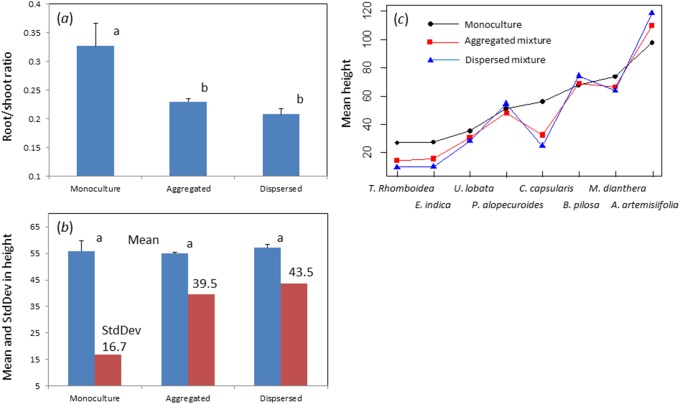
Biomass allocation and plant height variation for experiment I. (*a*) The mean root/shoot ratio varied with heterospecific interactions from monocultures, aggregated to dispersed mixture plots. (*b*) The within-plot mean (left bars) and standard deviation (right bars) in plant height (in cm, calculated over all grids in each plot) versus heterospecific interactions. (*c*) The average height for each of the eight species varied from monocultures, aggregated mixtures to dispersed mixtures. Data bars for the root/shoot ratio in (*a*) and the mean height in (*b*) are mean+1 standard error. Bars with different letters above are significantly different at *P* = 0.05. The values for the standard deviation for height are shown in (*b*).

The root to shoot ratio of the plants in experiment I is shown in Fig. 4*a*. The ratio consistently decreased from monocultures, to aggregated and finally to dispersed mixtures. The ratio for the monoculture plots was significantly higher than that for the aggregated and dispersed mixture plots (*P*<0.05), although the latter two were not significantly different. These results indicated that significantly more biomass was allocated to the shoots than to the roots in mixture plots than in monocultures. Although plant height showed no significant difference among the three types of plots, the within-plot variance in height increased substantially in the dispersed plots (Fig. 4*b*). At the individual species level, it is obvious that short plants became shorter as we move from monocultures to aggregated and finally to dispersed mixtures, while tall plants tended to become taller along the gradient (Fig. 4*c*).

## Discussion

An inevitable outcome of any increase in species diversity is the increase in the frequency of heterospecific interactions. Understanding how heterospecific interactions affect productivity lies at the heart of the diversity-productivity studies. Niche differentiation, facilitation and frequency-dependent growth are among the major mechanisms used to explain the positive effects of biodiversity on ecosystem functioning [24], [7], [5], [13], [21], [18]. These mechanisms can enhance the utilization of soil nutrients [21], [22], [23] and reduce competition for resources [24], [25]. Central to the understanding of these mechanisms is how heterospecific neighborhood interactions promote the productivity of multispecies communities.

Our results showed that there was a strong residual effect of heterospecific neighbor interactions after the diversity effects had been taken into account and an equally strong residual diversity effect after the number of heterospecific neighbors had been taken into account for aboveground biomass in both experiments (Table 1). Similar results held for belowground biomass in experiment I although the effects of diversity and heterospecific interactions were quite weak. These results suggested that it was important to include both diversity and neighbor interactions when explaining the positive diversity-productivity relationship. For example, for the belowground biomass in experiment I, if the frequency of heterospecific interactions were excluded from model (1), the effect of richness would be extremely significant with *P* virtually being 0 (Fig. 2). The same interpretations applied to the results for aboveground biomass of both experiments where the effect of species richness on biomass for the analysis without inclusion of the heterospecific interaction term was significantly higher than that with the heterospecific term (*P*<0.001, log-likelihood ratio test). The inclusion of heterospecific interactions decreased the effect of species richness because species richness and the frequency of heterospecific interactions were highly correlated. Their correlations for experiment I was *R* ^2^ = 0.86 (high because there were only two species richness levels) and *R* ^2^ = 0.25 for experiment II (four richness levels). It is remarkable to note that the dispersed mixtures significantly enhanced the effect of species richness on productivity compared to the aggregated mixtures (Fig. 3), which provided unequivocal evidence for the importance of heterospecific interactions on productivity.

It is, however, worth noting that the effect of species richness is not entirely explained by the frequency of heterospecific interactions. There are three possible reasons why the richness effect is not amount to the effect of the frequency of heterospecific interactions. First, the frequency of heterospecific interactions depends on the spatial distribution of conspecifics within plot. For a given number of species in a plot, the frequency of heterospecific interactions is small for aggregated conspecifics compared to dispersed conspecifics. Second, because the frequency of heterospecific interactions is a simple count of neighborhoods, it does not necessarily represent the genuine competitive (or facilitative) interactions between heterospecific individuals. To measure the competitive (or facilitative) interactions, distance between neighbors has to be considered. Third, the interactive strength is different between individuals of different species, e.g., the neighborhood interaction between individuals of species A and B is not equivalent to the interaction between the individuals of species B and C. This “diffused” neighborhood interactions among species could also contribute to the difference in the residual effects between richness and the frequency of heterospecific interactions.

Both theoretical and empirical studies have shown that neighbor interactions play a major role in shaping community structure, species coexistence and individual performance [31], [32], [33] and thus are central to understanding complementarity effects [24], [21], [18], [25], [28], [29]. Hille Ris Lambers et al. [21] showed that heterospecific interactions are an important complementarity mechanism that results in a more efficient use of soil nitrate. The effect of heterospecific interactions could be both positive (facilitation by legume species) and negative (competition with C4 species) [21]. Our study also showed that heterospecific interactions that may not be due to belowground heterospecific interactions, but were a result of neighborhood competition for light, play key roles in enhancing community productivity. The results in Table 1 show that, in addition to heterospecific interactions, diversity also significantly contributed to increasing productivity.

Several mechanisms can explain the effect of neighbor interactions on diversity-productivity relationships. Our study suggested that in grassland communities, variation in plant height played an important role. Although there was little change in mean plant height, the variation in height increased substantially from monoculture to aggregated and finally to dispersed mixtures (Fig. 4*b*). This increase in height variation means that the vertical structure from monoculture to aggregated and dispersed mixtures became more complex and heterogeneous, which probably increased the efficient interception and utilization of light. This complexity in complementary space occupancy is predicted by theory and can occur both belowground [34] and aboveground [35]. Overyielding in mixture plots could occur if tall plants become taller while short plants become shorter in more dispersed mixture plots, compared to less dispersed mixtures or monoculture plots, and the reduced growth of the short plants in mixture plots is compensated by the increased growth of tall plants. This was observed in our experiments (Fig. 4*c*) and means that heterospecific interactions in mixture plots led to the differentiation in height growth and thus to a more efficient use of light resources. We would further hypothesize that heterospecific neighbor interactions will also make shallow-root species produce shallower roots and deeper root species produce deeper roots, thus optimizing the use of soil water and nutrients. Overyielding is achieved by compensating the growth loss in the shallow root species by the growth gain in the deeper root species. We conclude that no matter what complementarity mechanisms invoke the positive diversity-productivity relationship, a differentiation in plant growth (either aboveground or belowground or both) must be seen for the relationship to hold.

## Supporting Information

Table S1 **Data of experiment I.** The variables are self-evident.
(XLSX)

Table S2 **Data of experiment II.** The variables are self-evident.
(XLSX)
